# Overexpression of *eIF3D* in Lung Adenocarcinoma Is a New Independent Prognostic Marker of Poor Survival

**DOI:** 10.1155/2019/6019637

**Published:** 2019-12-07

**Authors:** Dan Wang, Yichen Jia, Wendan Zheng, Changzheng Li, Wen Cui

**Affiliations:** ^1^Institute of Forensic Medicine and Laboratory Medicine, Jining Medical University, Hehua Road 133#, Jining City, 272067 SD, China; ^2^Institute of Medical Technology, Qiqihar Medical University, Jianhua District, Qiqihar City, 161006 HLJ, China; ^3^The Second Clinical Medical College, Jining Medical University, Hehua Road 133#, Jining City, 272067 SD, China

## Abstract

The eukaryotic initiation factor 3 (eIF3) is the largest and most complex translation initiation factor in mammalian cells. It consists of 13 subunits and among which several were implicated to have significant prognostic effects on multiple human cancer entities. To examine the expression profiles of *eIF3* subunits and determine their prognostic value in patients with lung adenocarcinoma (LUAD), the genomic data, survival data, and related clinical information were obtained from The Cancer Genome Atlas (TCGA) project for a secondary analysis. The results showed that among ten aberrantly expressed *eIF3* subunits in tumours compared with adjacent normal counterparts (*p* < 0.05), only upregulated *eIF3D* could predict poor overall survival (OS) outcome independent of multiple clinicopathological parameters (HR = 2.043, 95% CI: 1.132-3.689, *p* = 0.018). Chi-square analysis revealed that the highly expressed *eIF3D* group had larger ratios of patients with advanced pathological stage (68/40 vs. 184/206, *p* = 0.0046), residual tumour (13/4 vs. 163/176, *p* = 0.0257), and targeted molecular therapy (85/65 vs. 138/164, *p* = 0.0357). In silico analysis demonstrated that the altered expression of *eIF3D* was at least regulated by both copy number alterations (CNAs) and the hypomethylation of cg14297023 site. In conclusion, high *eIF3D* expression might serve as a valuable independent prognostic indicator of shorter OS in patients with LUAD.

## 1. Introduction

Lung cancer is one of the most common and lethal diseases afflicting the global population. It is histologically divided into small cell lung cancer (SCLC) and non-small-cell lung cancer (NSCLC). Lung adenocarcinoma (LUAD) is the most common subtype of NSCLC, accounting for more than 40% of NSCLC cases and showing a clear upward trend in the past few decades [1]. The overall survival (OS) rate for LUAD patients remains low despite the advances in clinical treatment. Due to the insignificant early symptoms and the lack of specific markers, the early diagnosis of LUAD is extremely difficult, and patients usually have a stage of local progression or later when they are diagnosed, missing the best time of surgery [2–4]. Therefore, the search for new and effective biomarkers to screen out high-risk patients and predict prognosis of LUAD has become an important part of lung cancer prevention and treatment.

mRNA translation is a key step in the regulation of eukaryotic gene expression. Its dysregulation would result in abnormal gene expression and lead to uncontrolled cell growth, which potentially caused tumourigenesis [5]. The initiation of mRNA translation is the rate-limiting step during which translation regulation is primarily achieved and its dysregulation has received considerable attention, as well as aberrant expression of eukaryotic translation initiation factors (eIFs) in various cancer entities has been observed in a growing number of studies [6–14]. eIF3, the largest and most complex eIF in mammalian cells, consists of 13 nonidentical subunits (eIF3a–m). The main function of eIF3 has been demonstrated to promote nearly every step of translation initiation as a scaffold, including promoting the 43S preinitiation complex formation, stimulating mRNA binding with 43S preinitiation complex, scanning and recognizing AUG start codon, and dissociating the posttermination 80S ribosome by binding with 40S ribosomal subunit [15–18]. The altered expression of *eIF3* subunits has been found in a wide range of tumour entities, including breast cancer [19–21], gastric cancer [22], NSCLC [23], and liver cancer [24], supporting their high potential use as prognostic biomarkers and therapeutic cancer targets. To the best of our knowledge, no previous studies have examined the roles of overall *eIF3* family members in LUAD. In this study, using gene expression data and survival data of LUAD patients from The Cancer Genome Atlas (TCGA) project, we examined the expression profiles of *eIF3* subunits and determined their prognostic roles in LUAD, as well as the underlying mechanisms of *eIF3D* dysregulation.

## 2. Materials and Methods

### 2.1. Clinical Cohorts and RNA-Seq Data from TCGA

mRNA expression data, survival data, and related clinical information of patients with LUAD in TCGA project were downloaded from the UCSC Xena browser (https://xenabrowser.net/). Only patients who had primary tumours and had not received neoadjuvant therapy were included in this analysis. The genomic and phenotypic data were collected. Briefly, RNA-Seq data of *eIF3* subunit genes were extracted. The phenotypic data included sample types, histological types, age at initial pathologic diagnosis, gender, pathologic stage, radiation therapy, targeted molecular therapy, residual tumour status, canonical mutations in *KRAS/EGFR/ALK*, tobacco smoking history, OS status, and OS time (weeks). To explore the underlying mechanisms of *eIF3D* dysregulation, the corresponding somatic mutations, copy number alterations (CNAs), and DNA methylations of *eIF3D* were obtained. The DNA methylation status was examined by the Infinium Human Methylation 450 Bead Chip.

### 2.2. Statistical Analysis

Statistical analysis was performed using GraphPad Prism 8 (GraphPad Inc., CA, USA) and SPSS Statistics 20 (SPSS Inc., IL, USA) in this study. Comparison between two groups was performed using unpaired *t*-test with Welch's correction. The differences in clinicopathological parameters between LUAD patients with high or low gene expression were compared using the chi-square test by two-sided Fisher's exact test. Gene expression levels were categorized as low or high according to the median values. Kaplan-Meier curves of OS were generated using GraphPad Prism, and differences in survival rate between groups were analysed by log-rank test. Univariate and multivariate Cox regression models were utilized to assess the prognostic value of *eIF3* subunits regarding OS. Genes with a *p* value of <0.05 in log-rank test were subsequently included in univariate and multivariate Cox analyses. Forest plots for multivariate Cox regression models were also drawn by GraphPad Prism. Pearson correlation coefficients between *eIF3D* expression and its linear DNA copy number values in patients, as well as the methylation level of its CpG site cg14297023, were calculated to assess their correlations. *p* < 0.05 indicates statistically significant for all above statistical analyses.

## 3. Results

### 3.1. The mRNA Expression Levels of *eIF3* Subunits in LUAD Patients

**Figure 1:**
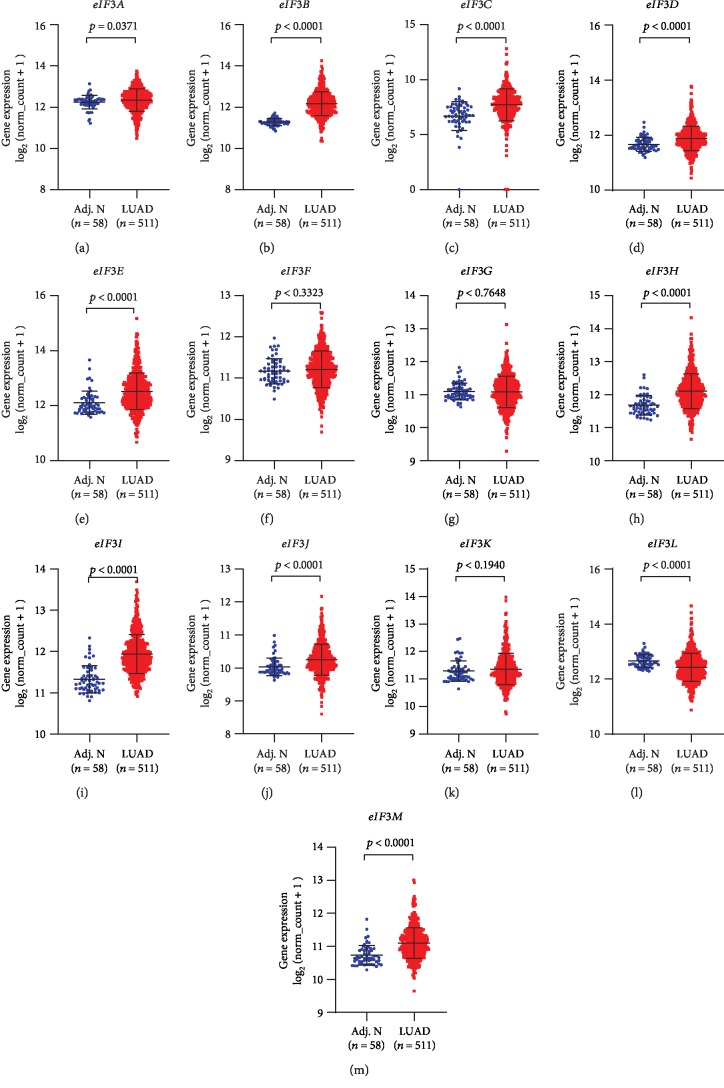
Plot charts showing mRNA expression of *eIF3* subunits in LUAD tissues and adjacent normal tissues. Adj. N: adjacent normal tissues.

Using RNA-Seq data in TCGA, the mRNA expression levels of all 13 *eIF3* subunits were compared between LUAD (*n* = 511) and adjacent normal tissues (*n* = 58). As shown in Figure 1, levels of nine *eIF3* subunits involving *eIF3A*, *eIF3B*, *eIF3C*, *eIF3D*, *eIF3E*, *eIF3H*, *eIF3I*, *eIF3J*, and *eIF3M* were significantly increased in cancer tissues, whereas the *eIF3L* expression level in tumours was independently decreased than that of normal tissues (*p* < 0.05). No significant variation was observed in *eIF3F*, *eIF3G*, and *eIF3K* levels between the two groups (*p* ≥ 0.05).

### 3.2. Association between *eIF3* Subunit Expression Levels and Clinical Features in LUAD Patients

Next, we summarized the association between gene expression levels of the 13 *eIF3* subunits and clinicopathological parameters of LUAD patients (Table 1). Chi-square analysis showed that five subunit genes associated significantly with a single clinicopathological variable, respectively, and among which *eIF3C* and *eIF3H* expression increased in high pathological stage (*eIF3C*: 64/44 vs. 188/207, *p* = 0.0388; *eIF3H*: 67/41 vs. 186/209, *p* = 0.0066), *eIF3F* and *eIF3L* expression increased in patients with ages no lower than 65 (*eIF3F*: 144/129 vs. 94/125, *p* = 0.0367; *eIF3L*: 151/122 vs. 93/126, *p* = 0.0050), while high expression of *eIF3G* was observed more in female patients (157/120 vs. 102/132, *p* = 0.0034).

Among the five genes whose expression levels were significantly associated with two clinicopathological variables or more, the high *eIF3A* expression group had larger proportion of male patients (134/100 vs. 120/157, *p* = 0.0019) and patients with smoking history (220/202 vs. 29/46, *p* = 0.0336), while the expression of the other four genes was consistently positively associated with high pathological stage (*eIF3B*: 66/42 vs. 189/206, *p* = 0.0168; *eIF3D*: 68/40 vs. 184/206, *p* = 0.0046; *eIF3E*: 64/44 vs. 186/209, *p* = 0.0297; *eIF3J*: 63/45 vs. 183/212, *p* = 0.0299). Besides, the more highly expressed *eIF3B* was also discovered in patients with ages lower than 65 (126/93 vs. 122/151, *p* = 0.0050), radiation therapy (37/21 vs. 194/202, *p* = 0.0483), and targeted molecular therapy (87/63 vs. 143/159, *p* = 0.0361). The high *eIF3D* expression group had a significantly larger ratio of patients with residual tumour (13/4 vs. 163/176, *p* = 0.0257) and targeted molecular therapy (85/65 vs. 138/164, *p* = 0.0357). And high expression of *eIF3E* and *eIF3J* was observed more in patients with residual tumours (13/4 vs. 172/170, *p* = 0.0456) and without mutations in *KRAS*/*EGFR*/*ALK* (47/48 vs. 86/46, *p* = 0.0206), respectively. No significant association was observed between *eIF3I*, *eIF3K*, *eIF3M*, and other above-mentioned clinical features.

### 3.3. The Prognosis Value of *eIF3* Subunit Expression in LUAD Patients

**Figure 2:**
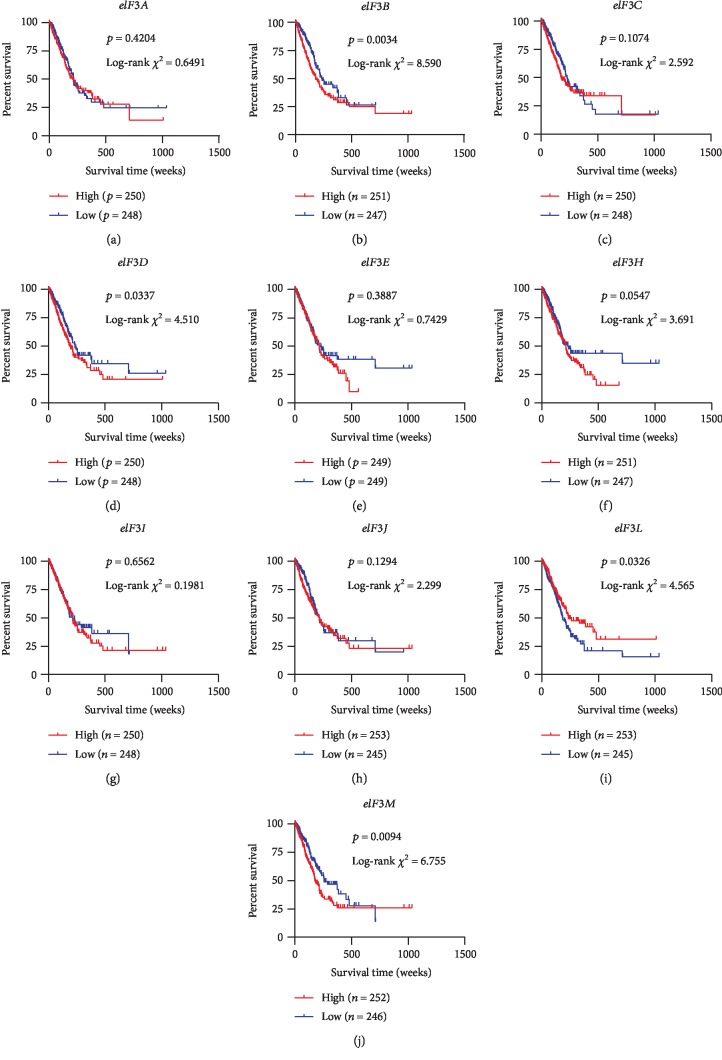
Kaplan-Meier survival analysis of differentially expressed subunits of *eIF3* for OS in LUAD patients. *χ*^2^ and *p* values assessed by log-rank test and number of patients in each group are provided in the figure.

Since we observed that ten *eIF3* subunits were significantly aberrantly expressed in tumours compared with adjacent area, we next evaluated the association between their expression levels and the survival outcomes of LUAD patients via Kaplan-Meier survival analysis (Figure 2). The results showed that patients with high expression levels of *eIF3B*, *eIF3D*, or *eIF3M* exhibited worse prognosis than those with low expression levels (*p*_eIF3B_ = 0.0034, *p*_eIF3D_ = 0.0337, and *p*_eIF3M_ = 0.0094). On the contrary, the elevated expression of *eIF3L* predicted better prognosis for our studied patients with LUAD (*p* = 0.0326). No significant differences in prognosis were observed between patients with high and low *eIF3A*, *eIF3C*, *eIF3E*, *eIF3H*, *eIF3I*, and *eIF3J* expression levels.

**Figure 3:**
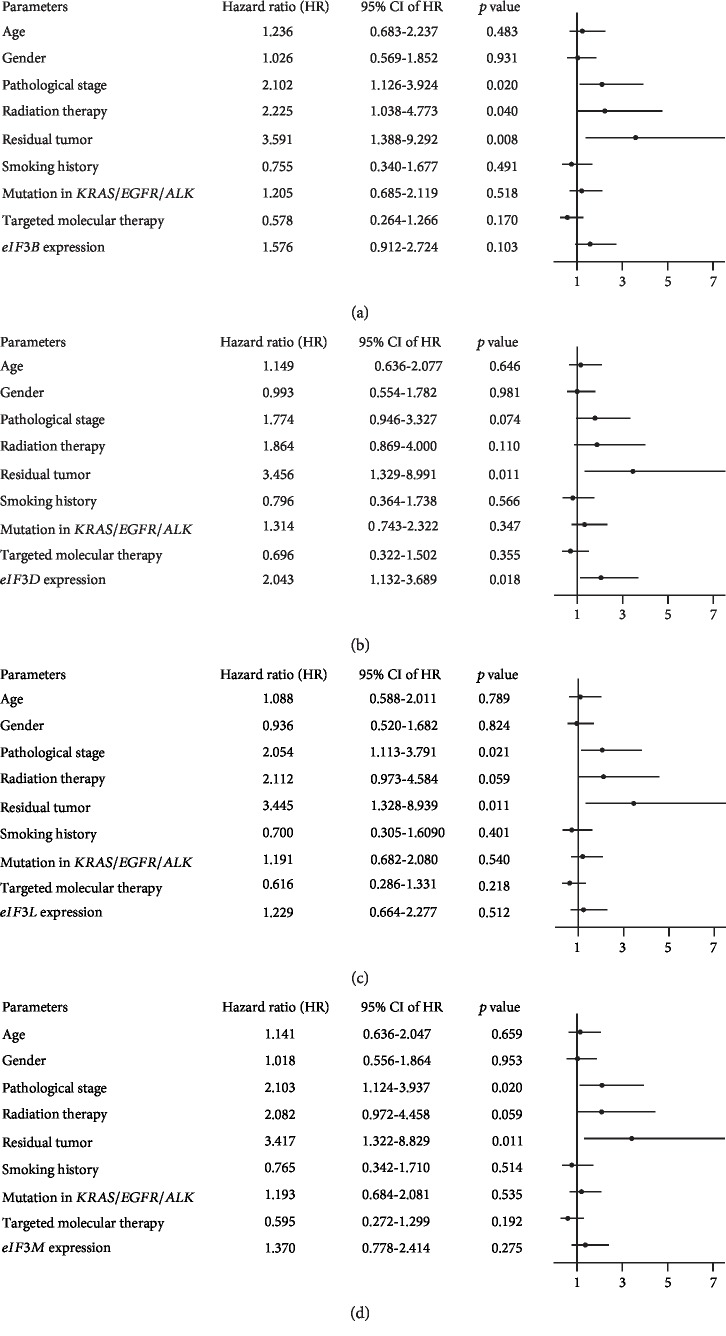
Forest plots for multivariate Cox regression models of four *eIF3* subunits: (a) *eIF3B*; (b) *eIF3D*; (c) *eIF3L*; (d) *eIF3M*.

To verify the robust prognostic roles of *eIF3B*, *eIF3D*, *eIF3L*, and *eIF3M*, the univariate and multivariate Cox regression analyses were performed. Univariate analysis showed that besides the expression levels of the four genes (HR_eIF3B_ = 1.563, *p* = 0.004; HR_eIF3D_ = 1.377, *p* = 0.034; HR_eIF3L_ = 0.710, *p* = 0.023; and HR_eIF3M_ = 1.521, *p* = 0.006), pathological stage (HR = 2.581, *p* < 0.001), radiation therapy (HR = 2.016, *p* < 0.001), and residual tumour (HR = 4.187, *p* < 0.001) were also significant prognostic factors (Table 2). By running four separate multivariate models (*eIF3B*, *eIF3D*, *eIF3L*, and *eIF3M*) adjusted by a series of clinicopathological parameters, it demonstrated that *eIF3D* expression was an independent risk factor for OS (HR = 2.043, 95% CI: 1.132-3.689, *p* = 0.018, Figure 3(b)), while no significant correlations were observed between other three genes and the OS (Figures 3(a), 3(c), and 3(d)). This result indicated that the overexpression of *eIF3D* could independently predict poor prognosis for patients with LUAD.

### 3.4. In Silico Analysis of the Potential Mechanisms Underlying *eIF3D* Upregulation in LUAD Patients

To explore the mechanisms of *eIF3D* dysregulation in patients with LUAD, we examined the correlations between *eIF3D* mRNA expression and its genetic (typically somatic mutations and CNAs) and epigenetic (typically methylation) alterations using the corresponding data in TCGA. Results showed that among 543 cases with somatic mutations measured, only three missense mutations were observed (data not shown); thus, we did not make further statistical analysis between *eIF3D* expression and its somatic mutations for the insufficient number of mutation cases.

**Figure 4:**
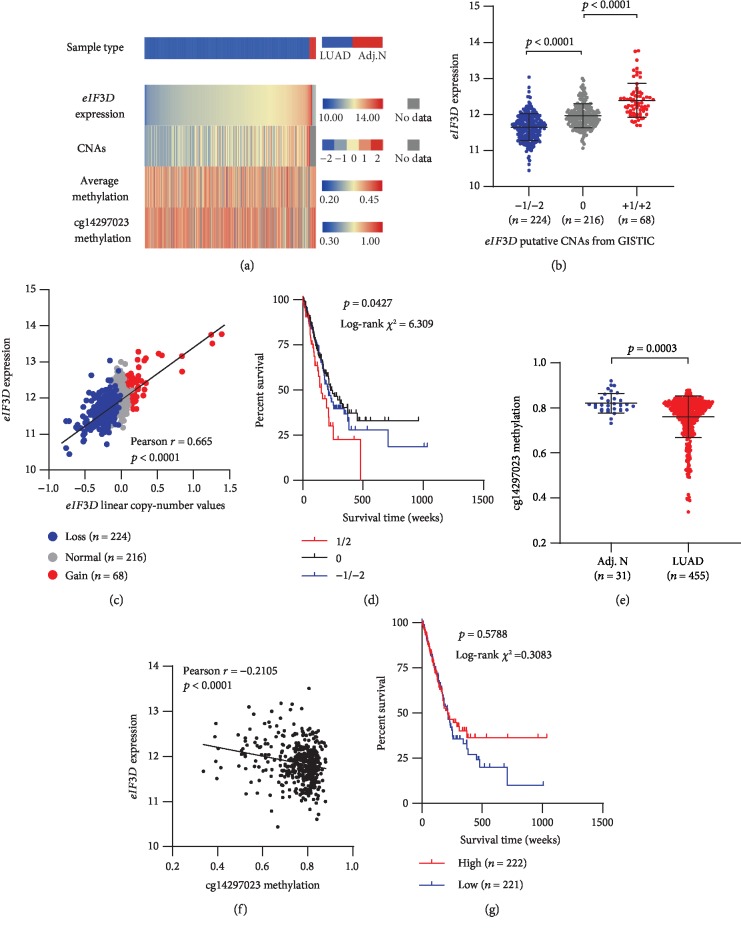
Copy number alterations and DNA methylation analysis of *eIF3D* in LUAD patients. (a) Heatmap showing *eIF3D* expression and corresponding DNA copy number alterations, averaged DNA methylation, and methylation in cg14297023. (b) Plot chart showing *eIF3D* expression in different CNA groups. (c) Linear regression analysis of the correlation between *eIF3D* expression and its linear copy number values. (d) Kaplan-Meier survival analysis of different *eIF3D* CNA levels for OS in patients with LUAD. (e) Plot chart showing DNA methylation of cg14297023 site in LUAD tissues and adjacent normal tissues. (f) Linear regression analysis of the correlation between cg14297023 methylation and *eIF3D* expression. (g) Kaplan-Meier survival analysis of different methylation levels in cg14297023 for OS in patients with LUAD. Adj. N: adjacent normal tissues; GISTIC: Genomic Identification of Significant Targets in Cancer [25].

Among 508 cases with DNA CNAs identified, 68 cases (13.4%) had CNA gains (+1/+2), 224 cases (44.1%) had CNA losses (-1/-2), and 216 cases (42.5%) were copy neutral (0). Unpaired *t-*test analysis showed both CNA gains and losses had significant influence on *eIF3D* expression (*p* < 0.0001, Figure 4(b)) and that a strong correlation was also observed between *eIF3D* expression and its linear copy number values by a regression analysis (Pearson *r* = 0.665, *p* < 0.0001, Figure 4(c)). Besides, Kaplan-Meier survival analysis showed that patients with CNA gains exhibited significantly worse prognosis than those with CNA losses and copy neutral (*p* = 0.0427, Figure 4(d)). As for *eIF3D* DNA methylation in LUAD, the methylation status of 23 CpG sites was measured by Methylation 450k, and among which nine were hypomethylated in tumour tissues (*n* = 455) compared with the adjacent counterparts (*n* = 31) ([Supplementary-material supplementary-material-1]). The regression analysis demonstrated a significantly negative correlation between *eIF3D* expression and a single CpG site of cg14297023 in tumour tissues (Pearson's *r* = −0.2105, *p* < 0.0001, Figure 4(f)), but no direct correlation was observed between cg14297023 methylation levels and the survival outcomes (*p* = 0.5788, Figure 4(g)). Overall, these results indicated that the expression of *eIF3D* was at least regulated by both CNAs and cg14297023 methylation.

## 4. Discussion

In this study, we examined the mRNA expression profiles of all 13 *eIF3* family members and determined their prognostic roles in LUAD patients using corresponding data from TCGA project. The integrated results demonstrated that the overexpression of *eIF3D* could independently predict poor prognosis for patients with LUAD.

Eukaryotic translation initiation depends on ribosomal subunits and, at least, 12 auxiliary proteins named eukaryotic initiation factors (eIFs), and among which eIF3 is the largest and most complex one comprising 13 subunits assembled together in an orderly way. Deregulation of *eIF3* expression and/or function has been proposed to play either a causal role or at least contribute to the etiology of various cancer entities. In our study, ten *eIF3* subunits were observed aberrantly expressed in LUAD tumour tissues than that of normal counterparts. Studies have revealed that imbalanced expression of single *eIF3* subunit may affect the overall expression profiles of the entire *eIF3* complex [26], which may explain why the vast majority of *eIF3* subunits were observed to be aberrantly expressed in our study. The misregulation of *eIF3* subunits may contribute to malignancy via several possible means. First, the altered expression of mammalian *eIF3* subunits, especially those in the core regions defined as octamer (a, c, e, f, h, k, l, m) and YLC (Yeast- Like-Core: b, g, i), may impact on the correct assembly of the entire eIF3 complex [26], leading to irregular mRNA translation and causing disease or its quick progression. Second, the overexpression of single *eIF3* subunit and/or the resulting upregulation of the entire eIF3 complex would induce a more efficient translation initiation rate of specific mRNAs, which is known as a common feature in cancer [27]. Third, besides translation initiation, the *eIF3* subunits may function in other important molecular events or cellular processes associated with a disease phenotype.

The eIF3D, a peripheral subunit attaching to the eIF3 holocomplex via eIF3E, showed very distinct characteristics with its other family members. It is the only subunit whose misregulation affects neither the expression of the other *eIF3* subunits nor the integrity of the eIF3 complex but is nonetheless essential for cell proliferation [26]. Instead of the canonical roles of eIF3 in general translation, the essential biological function of eIF3D lies in driving cap-dependent but eIF4E-independent expression of a specific subset of mRNAs encoding proteins with vital cellular roles [28, 29]. As a noncanonical cap-binding protein, eIF3D was also found to act as an indispensable assistant to help DAP5 which was supposed to initiate only IRES- (internal ribosome entry site-) mediated translation to promote a widespread alternate form of cap-dependent mRNA translation [30].

The overexpression of *eIF3D* has been observed to promote cell proliferation, migration, or/and tumour growth in several cancer entities, including ovarian cancer [31], renal cell carcinoma [32], gastric cancer (GC) [33], and gallbladder cancer (GBC) [34]. As being discovered in our study, all these high levels of *eIF3D* are associated with advanced tumour stage, indicating its potential role in tumour development. The *eIF3D* knockdown researches conducted in a series of cancer cell lines involving breast cancer [35], NSCLC [36], melanoma [37], acute myeloid leukaemia [38], and colon cancer [39] revealed that the deletion of *eIF3D* caused a significant reduction in cell proliferation and colony formation due to an arrest of cell cycle at G2/M phase, suggesting its key role in cell cycle control and apoptosis. Previous studies demonstrated that *eIF3D* affected cancer cell growth via multiple signaling pathways. Zhang et al. proposed that *eIF3D* exerted the tumour-promoting activities through GRK2-mediated activation of PI3K/AKT pathway in GBC [34]. Fan et al. found that knockdown of *eIF3D* inhibited the activation of Wnt/*β*-catenin signaling pathway in breast cancer cell lines via blockade of the expression of *β*-catenin, cyclin D1, and c-Myc [35]. By exploring the modifications of effector proteins in signaling pathways responsible for cell growth and apoptosis, the activations of three cancer-related molecules—AKT, HSP27, and SAPK/JNK—were found to be reduced by *eIF3D* knockdown in NSCLC cells [36]. Moreover, a series of phosphorylation upregulation was observed along with downregulation of *eIF3D* in colon cancer cells, including AMPK*α*, Bad, PRAS409, SAPK/JNK, and GSK3*β*, as well as the cleavage of PARP [39]. These mechanisms would help to explain the oncogenic properties of *eIF3D* in patients with LUAD. In our study, the prognostic value of *eIF3D* was confirmed in LUAD independent of multiple clinicopathological parameters. Being consistent with our findings, the strong correlations between high *eIF3D* expression and poor OS outcomes were also observed in patients with GC [33] and GBC [34], supporting its high potential role to serve as a useful prognostic marker and therapeutic target for the treatment of these cancers.

In this study, by assessing the potential mechanisms underlying *eIF3D* upregulation in LUAD, we found that the *eIF3D* dysregulation was partly regulated by the CNAs, as well as the hypomethylation of cg14297023 site which locates in the 3′UTR region of *eIF3D* gene according to the MethHC database (http://methhc.mbc.nctu.edu.tw/php/index.php) [40]. It is clear that a CNA is generally positively associated with the expression level of its corresponding gene. However, as for the role of DNA methylation in the 3′UTR on the influence of gene expression, far less is known than a comprehensive understanding of that in the gene promoter region. Recently, McGuire et al. proposed an explanation that DNA methylation in 3′UTR might control gene expression via two potential ways [41]. First, it may influence gene expression by increasing binding of proteins with methylation-binding domains or inhibiting other special protein binding or mask sequence recognition [42, 43]. Another potential explanation is that if different lengths of the 3′UTR are dependent on methylation, transcripts with shorter 3′UTRs would have greater mRNA stability and thereby higher gene expression [44]. On the contrary, studies also found that gene body methylation would reflect as a consequence of higher gene expression, rather than as a cause [45]. Therefore, the specific role of hypomethylation of cg14297023 site on *eIF3D* expression in LUAD cannot be concluded yet based on these hypotheses. Beyond that, we could not exclude other genetic or epigenetic mechanisms influencing the transcription and translation of *eIF3D*. Thus, experimental studies are required to further explore the role and other potential mechanisms of *eIF3D* in LUAD.

With regard to other subunits of *eIF3*, their misregulation and potential roles in tumour progression or survival outcome have been observed in a vast range of cancer entities (reviewed by [18, 46, 47]). In our study, even though their independent prognostic roles were not observed, we still got some noteworthy results. A previous study by Cattie et al. has revealed that loss-of-function mutations in *eIF3L* gene resulted in a 40% extension in lifespan in *Caenorhabditis elegans*, indicating its pivotal functional role in the regulation of cellular and organismal responses to aging [48]. By examining the links between gene expression levels and clinical features, the highly expressed *eIF3L* was found positively associated with LUAD patients with ages no lower than 65 (151/122 vs. 93/126, *p* = 0.0050, Table 1) in our study, adding to the evidence of its regulatory role during aging, as Cattie et al. proposed [48]. As an associated factor locating at the more peripheral position of the eIF3 complex, eIF3J is known to be weakly and unstably interacted with the complex and most likely plays only a supportive role in the overall translational efficiency in human cells [49]. However, little is known in the relation of *eIF3J* and carcinogenesis by now [15, 50, 51]. The mutations in *KRAS/EGFR/ALK* are the most common “driver” mutations detected in LUAD and are considered to play pivotal roles in carcinogenesis at multiple levels, thus acting as important genomic-guided therapeutic targets in LUAD (reviewed by [3]). In our study, the discovery of different expression levels of *eIF3J* occurring in patients with or without mutations in *KRAS/EGFR/ALK* (86/46 vs. 47/48, *p* = 0.0206, Table 1) may provide a clue of its potential impact on LUAD carcinogenesis accompanied by *KRAS/EGFR/ALK* genomic alterations. Besides, nine *eIF3* subunits beyond *eIF3D* were found aberrantly expressed in LUAD tissues and in which the expression levels of five subunits (*eIF3B*, *eIF3C*, *eIF3E*, *eIF3H*, and *eIF3J*) increased in LUAD patients with high tumour stage, implicating their roles in the maintenance or progression of LUAD. Therefore, it is meaningful to further explore the potential roles and underlying molecular mechanisms of action of these *eIF3* subunits as well as *eIF3D* in LUAD in the future.

## 5. Conclusions

High *eIF3D* expression might serve as a valuable independent prognostic indicator of shorter overall survival in patients with LUAD.

## Figures and Tables

**(a):** 

Parameters	*n* = 511		*eIF3A* expression		*p* value	*eIF3B* expression		*p* value	*eIF3C* expression		*p* value	*eIF3D* expression		*p* value
			High (*n* = 254)	Low (*n* = 257)		High (*n* = 258)	Low (*n* = 253)		High (*n* = 256)	Low (*n* = 255)		High (*n* = 258)	Low (*n* = 253)	
Age (years, mean ± SEM)	<65	219	105	114	0.5870	126	93	0.0050^∗^	103	116	0.3195	108	111	0.7856
	≥65	273	138	135		122	151		141	132		139	134	
	No data	19												
Gender	Females	277	120	157	0.0019^∗^	132	145	0.1830	137	140	0.7902	139	138	>0.9999
	Males	234	134	100		126	108		119	115		117	117	
Pathological stage	I/II	395	189	206	0.1285	189	206	0.0168^∗^	188	207	0.0388^∗^	184	206	0.0046^∗^
	III/IV	108	61	47		66	42		64	44		68	40	
	No data	8												
Residual tumour	R0	342	160	182	0.6294	173	169	>0.9999	169	173	>0.9999	163	176	0.0257^∗^
	R1/R2	17	9	8		9	8		8	9		13	4	
	RX/no data	152												
Smoking history	1	75	29	46	0.0336^∗^	32	43	0.1679	34	41	0.3807	41	34	0.3807
	2/3/4/5	422	220	202		219	203		217	205		205	217	
	No data	14												
Radiation therapy	No	396	204	192	0.1616	194	202	0.0483^∗^	196	200	>0.9999	188	208	0.1232
	Yes	58	24	34		37	21		29	29		34	24	
	No data	57												
Targeted molecular therapy	No	302	146	156	0.2308	143	159	0.0361^∗^	154	148	0.4245	138	164	0.0357^∗^
	Yes	150	82	68		87	63		70	80		85	65	
	No data	59												
Mutations in *KRAS/EGFR/ALK*	No	132	68	64	0.8935	65	67	0.1790	65	67	0.4230	78	54	0.3455
	Yes	95	50	45		38	57		52	43		50	45	
	No data	284												

**(b):** 

Parameters	*n* = 511	*eIF3E* expression		*p* value	*eIF3F* expression		*p* value	*eIF3G* expression		*p* value	*eIF3H* expression		*p* value	*eIF3I* expression		*p* value
		High (*n* = 255)	Low (*n* = 256)		High (*n* = 253)	Low (*n* = 258)		High (*n* = 259)	Low (*n* = 252)		High (*n* = 257)	Low (*n* = 254)		High (*n* = 254)	Low (*n* = 257)	
Age (years, mean ± SEM)	<65	103	116	0.3652	94	125	0.0367^∗^	112	107	0.7861	111	108	0.7856	110	109	0.7856
	≥65	140	133		144	129		136	137		134	139		133	140	
	No data															
Gender	Females	141	136	0.6574	148	129	0.0622	157	120	0.0034^∗^	134	143	0.3749	141	136	0.5945
	Males	114	120		105	129		102	132		123	111		113	121	
Pathological stage	I/II	186	209	0.0297^∗^	189	206	0.1599	195	200	0.4484	186	209	0.0066^∗^	198	197	>0.9999
	III/IV	64	44		60	48		58	50		67	41		54	54	
	No data															
Residual tumour	R0	172	170	0.0456^∗^	178	164	0.6275	176	166	0.3280	178	164	0.2123	184	158	0.4592
	R1/R2	13	4		10	7		11	6		12	5		11	6	
	RX/no data															
Smoking history	1	35	40	0.7074	34	41	0.5315	44	31	0.1329	32	43	0.2107	42	33	0.2113
	2/3/4/5	209	213		210	212		206	216		215	207		202	220	
	No data															
Radiation therapy	No	190	206	0.3274	195	201	0.7789	200	196	0.8890	192	204	0.1616	191	205	0.8883
	Yes	32	26		27	31		30	28		34	24		29	29	
	No data															
Targeted molecular therapy	No	149	153	0.8417	153	149	0.3180	159	143	0.3181	148	154	0.7650	141	161	0.4841
	Yes	72	78		68	82		71	79		76	74		76	74	
	No data															
Mutations in *KRAS/EGFR/ALK*	No	78	54	0.1252	77	55	0.8913	64	68	0.2853	72	60	0.1723	81	51	0.4148
	Yes	66	29		57	38		53	42		61	34		53	42	
	No data															

**(c):** 

Parameters	*n* = 511	*eIF3J* expression		*p* value	*eIF3K* expression		*p* value	*eIF3L* expression		*p* value	*eIF3M* expression		*p* value
		High (*n* = 252)	Low (*n* = 259)		High (*n* = 255)	Low (*n* = 256)		High (*n* = 255)	Low (*n* = 256)		High (*n* = 254)	Low (*n* = 257)	
Age (years, mean ± SEM)	<65	102	117	0.2772	102	117	0.2772	93	126	0.0050^∗^	104	115	0.5865
	≥65	141	132		141	132		151	122		137	136	
	No data												
Gender	Females	129	148	0.1839	145	132	0.2488	144	133	0.2869	142	135	0.4778
	Males	123	111		110	124		110	124		112	122	
Pathological stage	I/II	183	212	0.0299^∗^	192	203	0.2792	191	204	0.4479	187	208	0.0505
	III/IV	63	45		59	49		57	51		63	45	
	No data												
Residual tumour	R0	165	177	0.8057	172	170	0.6211	170	172	0.6205	170	172	0.3208
	R1/R2	9	8		10	7		10	7		11	6	
	RX/no data												
Smoking history	1	36	39	0.9011	36	39	0.8031	39	36	0.7073	38	37	0.8024
	2/3/4/5	206	216		210	212		207	215		205	217	
	No data												
Radiation therapy	No	190	206	0.6734	191	205	0.3295	199	197	>0.9999	189	207	>0.9999
	Yes	30	28		32	26		29	29		28	30	
	No data												
Targeted molecular therapy	No	138	164	0.1099	154	148	0.3202	147	155	0.3181	141	161	0.5489
	Yes	81	69		69	81		81	69		75	75	
	No data												
Mutations in *KRAS/EGFR/ALK*	No	86	46	0.0206^∗^	70	62	>0.9999	71	61	0.1013	75	57	0.6855
	Yes	47	48		50	45		62	33		51	44	
	No data												

^∗^Indicates statistical significance (*p* < 0.05).

**Table 2:** Univariate analysis of OS in LUAD patients.

Parameters	Univariate analysis		
	HR	95% CI (lower-upper)	*p* value
Age			
<65 (*N* = 220)	1.000		
≥65 (*N* = 273)	1.145	0.849-1.545	0.375
Gender			
Female (*N* = 273)	1.000		
Male (*N* = 230)	1.030	0.768-1.382	0.843
Pathological stage			
I/II (*N* = 392)	1.000		
III/IV (*N* = 103)	2.581	1.884-3.535	<0.001^∗^
Radiation therapy			
No (*N* = 392)	1.000		
Yes (*N* = 58)	2.016	1.372-2.962	<0.001^∗^
Residual tumour			
R0 (*N* = 336)	1.000		
R1/R2 (*N* = 16)	4.187	2.333-7.512	<0.001^∗^
Smoking history			
1 (*N* = 72)	1.000		
2/3/4/5 (*N* = 417)	0.888	0.588-1.341	0.571
Mutations			
No (*N* = 125)	1.000		
Yes (*N* = 91)	0.866	0.558-1.346	0.523
Targeted therapy			
No (*N* = 299)	1.000		
Yes (*N* = 149)	1.159	0.830-1.620	0.386
*EIF3B* expression			
Low (*N* = 247)	1.000		
High (*N* = 251)	1.563	1.156-2.111	0.004^∗^
*eIF3D* expression			
Low (*N* = 248)	1.000		
High (*N* = 250)	1.377	1.024-1.854	0.034^∗^
*EIF3L* expression			
Low (*N* = 245)	1.000		
High (*N* = 253)	0.710	0.528-0.955	0.023^∗^
*EIF3M* expression			
Low (*N* = 246)	1.000		
High (*N* = 252)	1.521	1.127-2.053	0.006^∗^

^∗^Indicates statistical significance (*p* < 0.05).

## Data Availability

The data used to support the findings of this study are available from the corresponding author upon request.

## Supplementary Materials

Supplementary Materials Table S1: methylation status of eIF3D CpG sites in LUAD tissues and adjacent normal tissues.
