# Supplementary material for: Overexpression of eIF3D in Lung Adenocarcinoma Is a New Independent Prognostic Marker of Poor Survival
Source: Dis Markers. 2019 Dec 7;2019:6019637. doi: 10.1155/2019/6019637 (PMC6925810; doi:10.1155/2019/6019637)
Supplement: Supplementary Materials — Table S1: methylation status of eIF3D CpG sites in LUAD tissues and adjacent normal tissues. [file 6019637.f1.docx]

Table S1. Methylation status of *eIF3D* CpG sites in LUAD tissues and adjacent normal tissues.

| CpG sites | Methylation level in Adj. N (mean ± SD, n = 31) | Methylation level in LUAD (mean ± SD, n = 455) | Δ Mean | Differences in methylation between LUAD and Adj. N (*p* value) | Correlation with *eIF3D* (Pearson correlation coefficient) |
| --- | --- | --- | --- | --- | --- |
| cg04186249 | 0.7408 ± 0.0348 | 0.5939 ± 0.1240 | -0.1469 | < 0.0001 | -0.0250 |
| cg18984186 | 0.7420 ± 0.0250 | 0.6975 ± 0.0806 | -0.0445 | 0.0023 | -0.0155 |
| cg15606745 | 0.6718 ± 0.0416 | 0.5776 ± 0.0956 | -0.0942 | < 0.0001 | -0.0088 |
| cg06943853 | 0.7971 ± 0.0326 | 0.7901 ± 0.0734 | -0.0070 | 0.6000 | -0.0537 |
| cg04350355 | 0.7965 ± 0.0373 | 0.7687 ± 0.1077 | -0.0278 | 0.1545 | -0.0519 |
| cg04933168 | 0.6821 ± 0.0673 | 0.7181 ± 0.0997 | 0.0360 | 0.0482 | 0.0794 |
| cg13270055 | 0.4823 ± 0.0688 | 0.6447 ± 0.1141 | 0.1624 | < 0.0001 | 0.0270 |
| cg02031638 | 0.6208 ± 0.0511 | 0.6027 ± 0.1115 | -0.0181 | 0.3699 | 0.0298 |
| cg15367287 | 0.8091 ± 0.0386 | 0.7753 ± 0.0768 | -0.0338 | 0.0155 | 0.0056 |
| cg27158143 | 0.1808 ± 0.0648 | 0.1316 ± 0.0461 | -0.0492 | < 0.0001 | 0.0094 |
| cg23413685 | 0.0283 ± 0.0161 | 0.0276 ± 0.0173 | -0.0007 | 0.8223 | -0.0261 |
| cg11603096 | 0.0335 ± 0.0093 | 0.0359 ± 0.0127 | 0.0024 | 0.3163 | -0.0630 |
| cg18520856 | 0.0394 ±0.09267 | 0.0229 ± 0.0130 | -0.0165 | 0.0008 | -0.0931 |
| cg20351327 | 0.0242 ± 0.0318 | 0.0193 ± 0.0097 | -0.0049 | 0.0354 | -0.0073 |
| cg15768968 | 0.0151 ± 0.0074 | 0.0136 ± 0.0071 | -0.0015 | 0.2659 | -0.0707 |
| cg26819140 | 0.0213 ± 0.0066 | 0.0216 ± 0.0070 | 0.0003 | 0.8065 | -0.0369 |
| cg14240963 | 0.0378 ± 0.0189 | 0.0291 ± 0.0158 | -0.0087 | 0.8152 | 0.0269 |
| cg22833807 | 0.0883 ± 0.0160 | 0.0907 ± 0.0132 | 0.0024 | 0.3508 | 0.1961 |
| cg00332575 | 0.0874 ± 0.0190 | 0.0896 ± 0.0123 | 0.0022 | 0.3618 | 0.1887 |
| cg18454903 | 0.0676 ± 0.0145 | 0.0637 ± 0.0112 | -0.0039 | 0.0695 | -0.0219 |
| cg24530557 | 0.8328 ± 0.0525 | 0.8465 ± 0.0757 | 0.0137 | 0.3224 | 0.0945 |
| cg27575890 | 0.3378 ± 0.0323 | 0.4118 ± 0.0876 | 0.0740 | < 0.0001 | -0.0712 |
| cg14297023 | 0.8213 ± 0.0439 | 0.7607 ± 0.0922 | -0.0606 | 0.0003 | -0.2105 |

Red fonts indicate CpG sites which were significantly hypomethylated in tumor tissues than in the adjacent normal tissues. Adj. N: adjacent normal tissues; SD: standard deviation.
